# First Data on the Occurrence and Genotyping of *Enterocytozoon bieneusi* in Wrestling Camels in Türkiye

**DOI:** 10.1007/s11686-025-01061-8

**Published:** 2025-06-02

**Authors:** Neslihan Sursal Simsek, Irmak Cakmak, Emrah Simsek

**Affiliations:** 1 Department of Parasitology, Faculty of Milas Veterinary Medicine, Mugla Sitki Kocman University, Milas, Mugla 48200 Türkiye; 2 Department of Parasitology, Graduate School of Health Sciences, Aydin Adnan Menderes University, Aydin, 9020 Türkiye; 3 Department of Preclinical Science, Faculty of Milas Veterinary Medicine, Mugla Sitki Kocman University, Milas, Mugla 48200 Türkiye

**Keywords:** *Enterocytozoon bieneusi*, Genotyping, ITS, Camels, Türkiye

## Abstract

**Purpose:**

*Enterocytozoon bieneusi* is the most prevalent microsporidian parasite and has wide host diversity, including animals and humans. However, there are a limited number of studies on the presence, molecular epidemiology, and genotyping of *E. bieneusi* in camels worldwide. Currently, no data are available on the occurrence, distribution, and genotypes of *E. bieneusi* in wrestling camels in Türkiye. This study aimed to address the knowledge gap regarding *E. bieneusi* in wrestling camels in Türkiye.

**Methods:**

In this study, a total of 110 fecal samples from camels in various provinces of Türkiye were investigated to reveal the presence of *E. bieneusi*, and the subsequent zoonotic potential of isolates was assessed at the genotype level by PCR-sequence analysis of the ribosomal internal transcribed spacer (ITS) gene region.

**Results:**

Three fecal specimens were detected as *E. bieneusi* positive, and the overall prevalence was 2.72%. Further sequence analyses revealed a novel genotype hereby named camelEb from wrestling camels in the Aydin (*n* = 1) and Mugla (*n* = 2) provinces of Türkiye. In the phylogenetic analyses, the camelEb genotype clustered into group 6 with other genotypes reported from camels.

**Conclusion:**

This is the first report on the presence of *E. bieneusi* in wrestling camels, suggesting that camels could also serve as a potential reservoir or carrier for *E. bieneusi* in Türkiye.

## Introduction

Microsporidia are a highly diverse group of unicellular, spore-forming, obligate, and intracellular parasites closely related to fungi as a basal branch, or a sister group based on phylogenetic analyses [1–3]. These organisms have wide host diversity ranging from protists to vertebrates, including humans [4, 5]. To date, nearly 1700 microsporidian species belonging to 220 genera have been described according to morphological and molecular approaches, host-parasite relationships, and developmental cycles [1]. Seventeen microsporidia species have been reported from human cases, among which *Enterocytozoon bieneusi* is the most prevalent species, responsible for more than ~ 90.0% of human infections associated with wasting and diarrhea [1, 6]. Although the transmission pathways of *E. bieneusi* are not completely understood, the widespread occurrence of infectious spores in the environment and their ability to infect various animal kingdoms support the idea of zoonotic transmission [7]. The fecal-oral route is the primary pathway for transmission, where infection occurs through the consumption of water and food sources that have been contaminated with the spore stages of the pathogen [6], with some studies indicating that these contaminated sources are linked to outbreaks [8, 9]. Fumagillin is currently used to treat microsporidiosis; however, it causes thrombocytopenia, highlighting the need for more suitable therapeutic options [6]. Thus, to implement effective control strategies, it is essential to accurately identify the agent responsible for the disease [10]. Currently, PCR amplification and sequence analysis of the ribosomal internal transcribed spacer (ITS) gene region is still an accepted standard diagnostic approach for identifying *E. bieneusi*. Genotyping of this parasite relies on analysis of polymorphisms within the ITS region. For the genotyping of isolates, only 243 base pairs of the ITS region should be used, and the small-large subunit rRNA gene regions should be excluded [11–14]. The number of *E. bieneusi* genotypes is continually increasing, with more than 500 genotypes reported in humans, companion animals, livestock, wild mammals, birds, and water sources worldwide, validated through the analysis of sequence polymorphisms within the ITS gene region. *Enterocytozoon bieneusi* genotypes exhibit high diversity according to hereditary and genetic characteristics, and 11 major phylogenetic groups (groups 1 to 11) have been characterized based on ITS genotyping data [11]. In recent studies, new phylogenetic groups (groups 12 to 15) have been proposed according to comprehensive phylogenetic analysis [12, 14, 15]. Groups 1 and 2, which are the two major phylogenetic groups of *E. bieneusi*, have a broad range of hosts, including humans, and are attributed as zoonotic groups. In contrast, genotypes in groups 3 to 15 are genetically distinct from those groups, generally exhibiting host specificity and posing a minor or unknown public health risk [10, 11, 15, 16].

Although *E. bieneusi* has been detected in mammals, avian, and water sources worldwide (Table 1), data on its presence in camels are limited (Table 2). Camels have been used for a wide range of purposes, including transportation, agricultural activities, leisure, and production in different cultures, since they were domesticated around 3000–6000 years ago [17, 18]. The camel population is estimated to be approximately 41 million worldwide and is an important source of meat and milk, especially in the Asian and African populations [19, 20]. Two domestic camel species, namely *Camelus dromedarius* (one-humped, dromedary) and *C. bactrianus* (two-humped, bactrian), along with one wild camel species, *C. ferus*, have been recognized worldwide [17]. One-humped camels are well adapted to the deserts of Africa, Asia, and Australia, while two-humped camels are well adapted to the deserts of Central Asia [21]. In Türkiye, Bactrian x Dromedary F1 (Tülü) male hybrid camels are commonly bred for use in wrestling, which is organized during festivities that occur from November to April for many years [22, 23]. More recently, the emergence of camel farms to produce camel milk has been observed, highlighting the role of camels as a food source in addition to their cultural significance in Türkiye [23]. The camel population is estimated to be approximately 1197, with particularly prevalent populations located in the Aydin, Mugla, Antalya, Izmir, Denizli, Manisa, Canakkale, Balıkesir, and Mersin provinces in Türkiye [19, 24]. The dromedary camels distributed in 47 different countries account for approximately 95.0% of the Old-World camel population and have an important role in the economies of those countries [20, 25]. As previously mentioned, camels play a crucial role as a food source, especially in semi-arid and arid zones. Therefore, it is stated that the role of dromedaries has evolved from being the “ships of the desert” to becoming crucial “food security livestock” [20, 25]. In recent years, it has been emphasized that the camel industry has been developing and evolving to more large-scale production. Thereby camels may be important in the zoonotic transmission of some pathogens, especially in communities with inadequate sanitation and healthcare [20]. From 1970 to 2018, most (65.0%) of the publications related to zoonotic diseases reported from camels focused on Middle East respiratory syndrome, hydatidosis, brucellosis, and Rift Valley fever, as highlighted in a review by Zhu et al. [26]. *Echinococcosis* is the most studied disease among the zoonotic parasitic diseases in camels; however, *Cryptosporidium* spp., *Toxoplasma gondii*,* Trichinella* spp., *Fasciola* spp., and *Linguatula serrata* reported from camels are also regarded as significant potential public health hazards [26]. *Enterocytozoon bieneusi*, one of the important global public health problems worldwide, has also been reported from camels in recent years but limited to few studies [27–30]. A scientific gap related to the prevalence, distribution, genotypes, and zoonotic transmission potential of *E. bieneusi* in camels is still ongoing.

**Table 1:** The overall global prevalence of *E. bieneusi* in mammals, avian, and water sources

**Host species**	**Overall prevalence (%)**	**References**
Humans	6.59%	[31]
Cats	7.4%	[32]
Dogs	8.4%	[7]
Cattle	12.9%	[33]
Sheep	17.4%	[34]
Goats	16.3%	[34]
Birds	13.8%	[7]
Pigs	37.6%	[35]
Rodents	13.6%	[36]
Wild Boars	10.1%	[37]
Water Sources	58.5%	[7]

**Table 2:** The prevalence and genotypes of *E. bieneusi* in camels worldwide

Country	Host	Sample size (*n*)	Positive sample (*n*)	Prevalence (%)	Genotypes	References
Iran	Camel	30	4	13.3%	BEB6, Macaque1, CHG3	[38]
Egypt	Camel	102	^ND^	^ND^	^ND^	[27]
Algeria	Camel	39	8	20.5%	Macaque1, Camel-2,	[28]
China	Camel	407	122	30.0%	EbpC, EbpA, Henan-IV, BEB6, CM8, CHG16, O, WL17, CAM1 – CAM6	[29]
China	Camel	40	18	45.0%	CAM1, CAM2, BEB6	[30]
China	Camel	40	18	45.0%	^ND^	[39]
China	Camel	^NA^	^NA^	^NA^	CD7 CHS9	[40]
Türkiye	Camel	110	3	2.72%	camelEb	Present study

^NA^ Not Available; ^ND^ Not Detected

Therefore, in this study, we aimed to (1) investigate the occurrence and prevalence of *E. bieneusi* in wrestling camels, (2) determine *E. bieneusi* genotypes infecting camels, and (3) reveal phylogenetic relationships of detected genotypes with other validated genotypes worldwide.

## Materials and Methods

### Sampling and Parasitological Examination

**Fig. 1:**
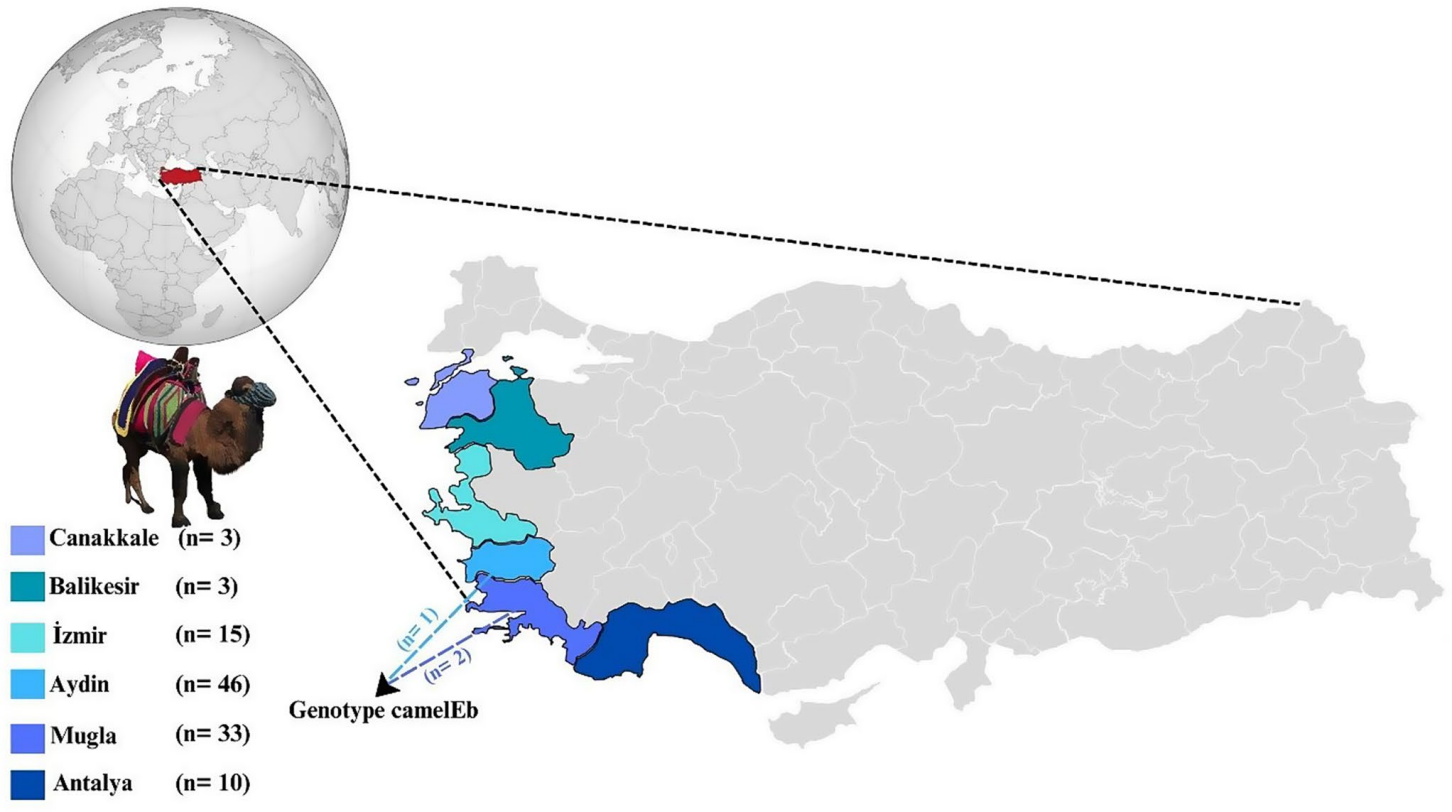
Sampling areas, number of fecal samples and *E. bieneusi* genotype characterized in camels in Türkiye

According to FAOSTAT data, Türkiye has a total of 1.197 wrestling camels, which are clustered in the provinces of Aydin, Denizli, Antalya, Mugla, Canakkale, Balikesir, Izmir, Mersin, and Manisa [19, 24]. Due to the cultural and economic importance of wrestling camels, obtaining owner consent for sampling presented a significant challenge and restricted access to certain regions. Nevertheless, approximately 10.0% of the total camel population of Türkiye was included in the sampling, providing meaningful epidemiological insights. For this purpose, between November 2023 and March 2024, a total of 110 fecal samples were collected from wrestling camels raised in Aydin (*n* = 46), Mugla (*n* = 33), Izmir (*n* = 15), Antalya (*n* = 10), Balikesir (*n* = 3), and Canakkale (*n* = 3) provinces of Türkiye (Fig. 1).

Fecal samples from each camel were promptly collected from fresh droppings on the ground using disposable gloves and placed individually into disposable sterile plastic containers. Age, sex, and region of camels were recorded. All the animals were healthy and presented no signs/clinical symptoms that could be attributed to any disease. The fecal specimens were then transferred to the parasitology laboratory via transport containers with ice and were immediately stored at 4 °C for further processing.

### DNA Extraction and PCR Amplification

To prevent environmental contamination, fecal balls from each camel were divided into two in a sterile petri dish; some amount of feces was taken from the interior of the fecal balls, homogenized, and used for genomic DNA (gDNA) extraction. gDNA from each fecal sample was individually isolated using the commercial DNA purification kit (QIAamp DNA Stool Mini Kit, Qiagen, Hilden, Germany) according to the manufacturer’s instructions. The isolated gDNA was stored at -20 °C until molecular analysis.

Polymerase chain reaction (PCR) was carried out to amplify the **~** 390 bp of the ITS gene region of *E. bieneusi* using the EBITS3/EBITS4 and inner primers EBITS1/EBITS2.4 [41]. The PCR reaction was carried out in a 25 µl final volume, comprising 12.5 µl of Dream Taq PCR Master Mix (Thermo Scientific, Waltham, MA, USA), 1 µM of each primer, and 10–30 ng of gDNA for the amplification of the target gene region. Amplification was performed using Applied Biosystems thermocycler (Thermo Scientific, Waltham, MA, USA). The thermal cycling conditions were as follows: an initial denaturation step at 94 °C for 2 min, followed by 35 cycles of 30 s at 95 °C, 30 s at 54 °C for the first round PCR, 51 °C for nested PCR, then 45 s at 72 °C, with a final extension step at 72 °C for 10 min. gDNA of the *E. bieneusi* isolate, which had been formerly confirmed by sequence analysis, was used as a positive control, and ultrapure DNAse-RNAse free water was used as a negative control during all PCR reactions. Amplicons (5 µl) were analyzed in 1.5% agarose gel, stained with SafeView^™^ (Applied Biological Materials, Richmond, BC, Canada), and visualized by UV illumination.

### Sequencing and Genotyping of *Enterocytozoon bieneusi* and Phylogenetic Tree Construction

PCR products were purified and sequenced in both directions (Macrogen, Amsterdam, The Netherlands) using the EBITS1/EBITS2.4 nested PCR primers for molecular characterization of isolates. The obtained ITS sequences were assembled and edited using the Geneious Prime 2024.0.5 software (https://www.geneious.com). All sequences were individually assessed by evaluating the phred scores and chromatogram quality, and a consensus sequence was generated. To identify species, final nucleotide sequences were analyzed using the BLASTn plugin in Geneious Prime. Afterward, the ITS sequences of 112 *E. bieneusi* genotypes belonging to 15 distinct phylogenetic groups [15] were downloaded from GenBank to create the sequence data set, including the novel sequence characterized in this study. Considering the *E. bieneusi* genotyping nomenclature [13], only the 243 base pairs of the ITS region were used, and the small-large subunit rRNA gene regions were excluded from the alignment data set. All sequences of the ITS region of genotypes were aligned using the MUSCLE [42] plugin available in Geneious Prime.

The phylogenetic tree was constructed using the Neighbor-Joining (NJ) algorithm with the Kimura 2-parameter model through the MEGAX [43] platform, employing 1000 bootstrap replicates. Genetic similarities among the genotypes included in the data set were calculated using the MUSCLE [42] alignment through the plugin available in Geneious Prime. The ITS nucleotide sequence of an *E. bieneusi* isolate has been submitted to GenBank with the following isolate name (TREbiencamel), genotype name (camelEb), and accession number (PQ665280).

## Results

### Prevalence of *E. bieneusi*

*E. bieneusi* positivity was detected in 3 out of 110 gDNA of camel fecal samples by nested PCR analysis of the ITS gene region, with an overall prevalence of 2.72%. The overall infection rates of *E. bieneusi* in fecal samples collected from Aydin and Mugla provinces were 2.17% (1/46) and 6.06% (2/33), respectively. The correlation between infection rate and age or sex could not be statistically evaluated as the age distribution of the animals was not suitable to form meaningful groups, and all camels included in this study were male.

### Genotyping and Phylogenetic Analyses of *E. bieneusi* Genotypes

Three purified nested PCR products of *E. bieneusi* positive specimens were successfully sequenced, and all sequences obtained from three isolates were wholly identical.

BLASTn analyses revealed that the ITS sequence (352 bp) of isolates found in this study showed 98.9% identity with CAM1 (MG602791), CAM2 (MG602792), and CAM4 (MG602794) genotypes of *E. bieneusi* reported from *C. bactrianus* [29]. Also, our isolate exhibited similarity with Macaque1 (LC270273–280) genotypes reported from *C. dromedarius* [28], ranging from 98.0 to 98.3%. Furthermore, it also showed 98.2% and 97.6% identity with CAM1 (MK843237) [30] and Macaque1 (PQ137025) genotypes reported from the camels, respectively.

MUSCLE alignment results of all the ITS (243 bp, reference gene region for *E. bieneusi* genotyping) sequences included in the dataset revealed that our isolate showed identity ranging from 97.5 to 97.9% with CAM1 (MG602791; MK843237), CAM2 (MG602792), and CAM4 (MG602794) genotypes reported from the camels. Additionally, it exhibited identity ranging from 97.1 to 97.5% with Macaque1 (PQ137025; LC270273–280) genotypes reported from the camels.

**Fig. 2:**
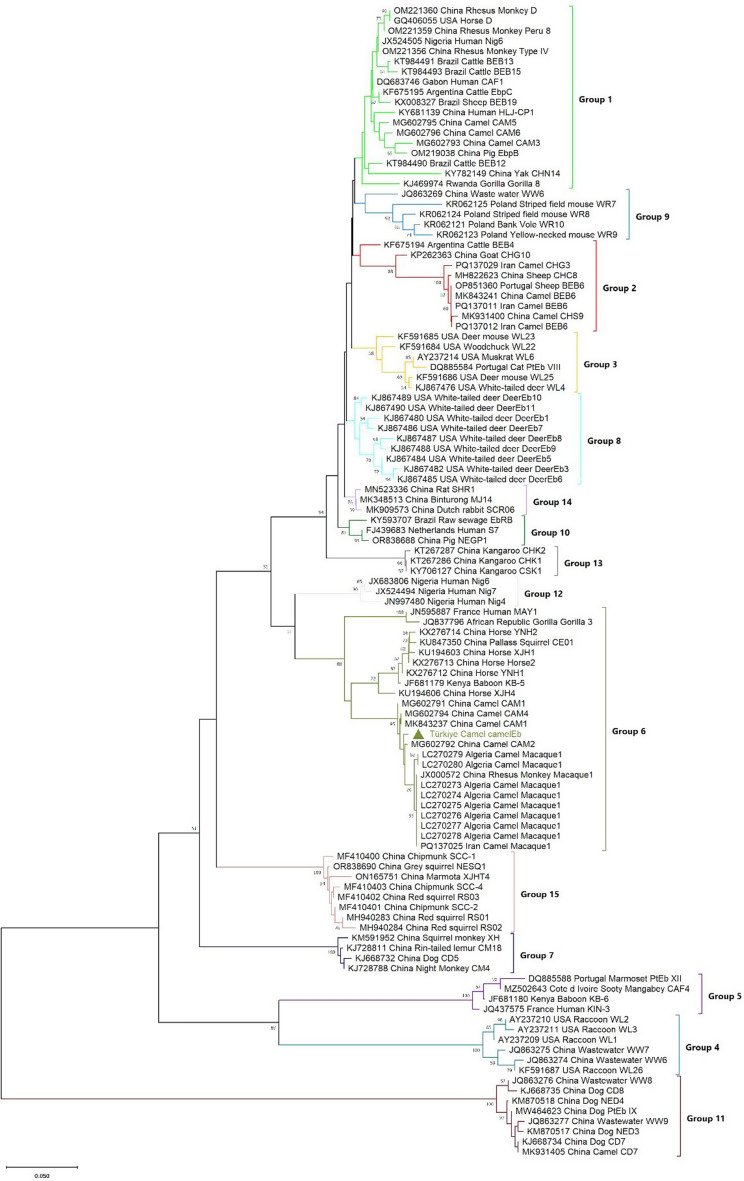
The evolutionary relationships of *Enterocytozoon bieneusi* genotypes inferred from ITS nucleotide sequences. Neighbor-Joining analysis based on Kimura two-parameter model was used the phylogenetic analyses of the ITS data set. Bootstrap values greater than 50.0% from 1000 pseudoreplicates were displayed. A novel *E. bieneusi* genotype identified in this study was indicated with triangle. The sequences are provided along with their GenBank accession numbers, the country of origin, host, and corresponding genotype names. Groups 12 to 15 are suggested by Jiang et al. [15] as potentially valid phylogenetic groups

These analyses revealed a novel genotype named camelEb from wrestling camels in the Aydin (*n* = 1) and Mugla (*n* = 2) provinces of Türkiye. The NJ tree, including the camelEb and the previously characterized genotypes from camels, and other hosts in GenBank, is presented in Fig. 2 with group branches robustly supported by bootstrap values above 50.0%. In the NJ tree, the novel camelEb genotype identified in this study and other genotypes reported from camels were clustered into group 6. Also, the camelEb genotype and genotypes found in camels were separated from other genotypes belonging to group 6 in the NJ tree. The novel camelEb genotype showed a genetic distance of 7.4–10.3% from the other genotypes clustered in group 6.

## Discussion

*Enterocytozoon bieneusi* is one of the most significant zoonotic microsporidian worldwide. It is primarily transmitted via the fecal-oral route and can infect a wide range of hosts. As summarized in Table 1, E. *bieneusi* has been reported in humans (6.59%) [31], companion animals such as cats (7.4%) [32] and dogs (8.4%) [7], as well as in various livestock species including cattle (12.9%) [33], sheep (17.4%), and goats (16.3%) [34]. Additionally, notable prevalence rates have been detected in birds (13.8%) [7], pigs (37.6%) [35], rodents (13.6%) [36], and wild boars (10.1%) [37]. The highest detection rate has been reported in water sources (58.5%) [7], highlighting the role of environmental contamination in the transmission cycle of this pathogen. These findings emphasize the importance of adopting a One Health approach to better understand the epidemiology of *E. bieneusi*.

To date, studies investigating the presence, molecular epidemiology, and genotyping of *E. bieneusi* in animal hosts in Türkiye remain limited. Reported prevalence rates include 2.7% in water buffaloes, 5.5% in domestic cats, 3.5% in pet budgerigars, 7.3% in chickens, 8.0% in sheep, 14.0% in tumbler pigeons, 18.7% in horses, and 19.3% in cattle, highlighting the wide host range of this pathogen [44–51].

There is currently no report on the presence and genotyping of *E. bieneusi* in camels in Türkiye. Recently, Sazmand et al. [20] have highlighted concerns about camels serving as potential reservoirs for zoonotic pathogens, particularly in communities with limited access to sanitation and healthcare. In Türkiye, Bactrian x Dromedary F1 (Tülü) male hybrid camels are generally bred for use in wrestling and festivities to maintain a cultural heritage. Camels are not just seen as animals but can serve as integral parts of their owners’ lives, viewed as symbols of status, companionship, or even family. That is, camels are in close contact with their owners. Therefore, research on zoonotic pathogens of camels, which are closely related to public health, is crucial. This study is the first report on the occurrence, prevalence (2.72%), and genotypes of *E. bieneusi* in fecal samples collected from wrestling camels across various provinces in Türkiye. Few studies conducted on the fecal samples of camels (*C. bactrianus* and *C. dromedarius)* in China [29, 30] and Algeria [28] revealed an overall *E. bieneusi* prevalence of 30.0% (122/407), 45.0% (18/40), and 20.5% (8/39), respectively. Qi et al. [29] linked the high prevalence (30.0%) in bactrian camels in China to factors such as free feeding and drinking water, and shared grazing with cattle, goats, sheep, and other livestock, as well as inadequate veterinary care. In another study [27], 102 fecal samples collected from dromedary camels with diarrhea (*n* = 26) and without diarrhea (*n* = 76) were screened for the presence of *E. bieneusi*. However, no positivity was detected. The authors considered that dromedary camels are not relevant hosts for the transmission of this pathogen in Egypt. Compared to the former limited number of studies [28–30], a low prevalence (2.72%) was detected in this study. As mentioned above, since camels are bred for hobby and cultural events in Türkiye, their owners take care of camels carefully, supply quality drinking water, and keep them in individual shelters. In addition to this, camels are not grazed in pastures and areas where different animals are grazed. So, the low prevalence of *E. bieneusi* in wrestling camels in Türkiye might be associated with improved care and feeding conditions.

Until today, some *E. bieneusi* genotypes have been recognized across a range of animal species in various provinces of Türkiye, including D and Type IV in cats [46], ERUSS1–4 in cattle [50], ERUH2–ERUH7, ERUSS1, BEB6 in horses [49], ERUSS1 and ERUNT1 in chickens [51], Peru6 in pigeons [47], TURKM1and N in budgerigars [48], YNDCEB-90 and J in water buffaloes [45], and BEB6 in sheep [44]. Another study [52] reported the TREb1–TREb6, ERUSS1, and BEB6 genotypes in raw milk sampled from water buffaloes, cattle, and sheep. Lastly, Type IV, BEB6, BEB8, and AEUEb genotypes have been reported from *Musca domestica* [53]. There have been no reports on the genotyping of *E. bieneusi* in camels in Türkiye, with a very limited number of studies on genotyping of *E. bieneusi* in camels worldwide. Recently, BEB6, Macaque1, CAM1–CAM6, EbpC, EbpA, Henan-IV, BEB6, CM8, CHG16, O, WL17, CD7, CHS9 and CHG3 genotypes have been reported in a few studies conducted on fecal samples collected from camels in China and Algeria [28–30, 38, 40]. The host-specific genotype CAM1 has been determined as the most predominant genotype in Bactrian camels in Xinjiang, China, while the second-most predominant genotype was EbpC [29]. Qi et al. [29] also highlighted the EbpC genotype, which fell into the human-pathogenic group 1, has zoonotic potential and concluded the bactrian camels may act as potential reservoirs for *E. bieneusi* transmission to both humans and other animals. This study provides the first genotyping data on *E. bieneusi* in camels in Türkiye, with three specimens found to be *E. bieneusi* positive, and all sequences obtained from isolates being wholly identical. Our isolates showed ranging from 97.5 to 97.9% identity with host-specific CAM1 (MG602791; MK843237), CAM2 (MG602792), and CAM4 (MG602794) genotypes reported from the camels in China [29, 30] Therefore, based on ITS sequence analyses, a novel genotype was characterized and named camelEb in wrestling camels in the Aydin (*n* = 1) and Mugla (*n* = 2) provinces of Türkiye. Compared to genotypes reported from other animal hosts in Türkiye, the *E. bieneusi* detected in camels was limited to one distinct genotype with no detection of genotypes that have been previously reported. This might be related to the limited sample size from some provinces outside of Aydin and Mugla, good care/feeding conditions, or the fact that the camels are not in close contact with other animals. The camelEb genotype clustered in Group 6 with the CAM1 (MG602791; MK843237), CAM2 (MG602792), CAM4 (MG602794), and Macaque1 (PQ137025; LC270273–280) genotypes reported from camels in China and Algeria (Fig. 2). camelEb genotype showed a genetic distance of 7.4–10.3% from the other genotypes clustered in group 6 (Fig. 2).

In conclusion, our study provides the first data on the prevalence and genotyping of *E. bieneusi* in wrestling camels in Türkiye, leading to the characterization of a novel genotype named camelEb. In a current study [29], the identification of host-specific genotypes as well as zoonotic genotypes in camels has highlighted the potential role of camels in the zoonotic transmission of *E. bieneusi*. The identification of novel genotypes and the host-adapted genotypes in camels worldwide emphasizes the need for further detailed research to evaluate their potential importance on public health. Further research involving a broader range of domestic and wildlife animal species, as well as human populations, is essential to gain a deeper understanding of the epidemiology, transmission dynamics, and zoonotic risk potential of *E. bieneusi* in Türkiye.

## Data Availability

The ITS nucleotide sequence of an *E. bieneusi* isolate has been submitted to GenBank with the following isolate name (TREbiencamel), genotype name (camelEb), and accession number (PQ665280).
